# Supplementary material for: Histone deacetylase knockouts modify transcription, CAG instability and nuclear pathology in Huntington disease mice
Source: eLife. 2020 Sep 29;9:e55911. doi: 10.7554/eLife.55911 (PMC7581428; doi:10.7554/eLife.55911)

**CAG repeat lengths in the cohorts of mice used in this study**

**(A)** In the 2.5-month age group, different cohorts of mice were used for instability measurements and for immunohistochemistry (IHC). In the 5-month and 10-month age groups instability and IHC were performed on the same mice.

**(B)** Mice used for IHC cohort were a subset of those used for instability measurements.

**(C)** A cohort of 5-month *Htt*^Q111/+^ mice harboring wild type *Hdac2* and *Hdac3* alleles, either with or without the *DARPP-32 Cre* transgene (*D9-Cre*). Error bars show SD.


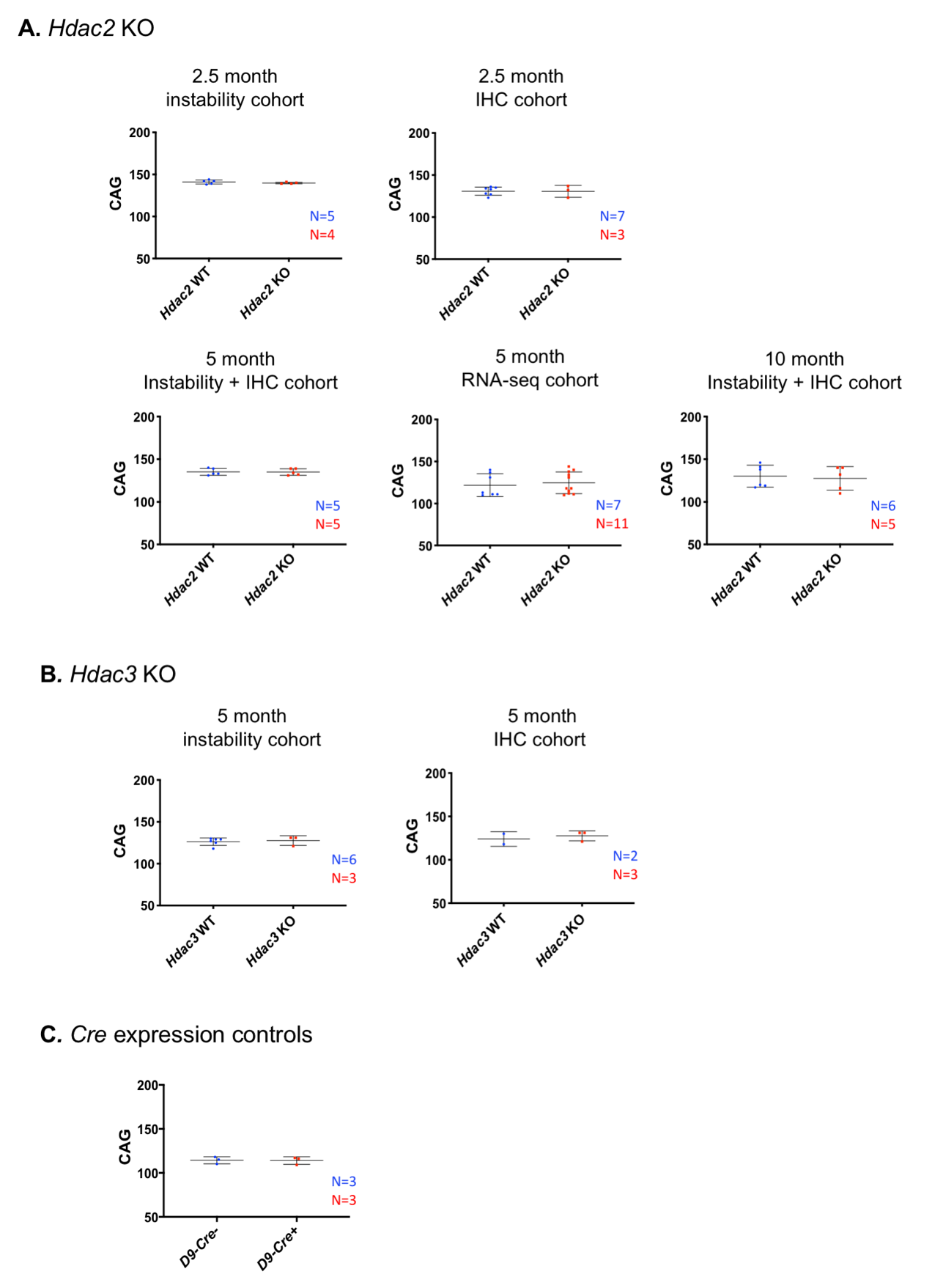

Supplement: Source data 1. [file elife-55911-data1.docx]
