# Supplementary material for: Histone deacetylase knockouts modify transcription, CAG instability and nuclear pathology in Huntington disease mice
Source: eLife. 2020 Sep 29;9:e55911. doi: 10.7554/eLife.55911 (PMC7581428; doi:10.7554/eLife.55911)
Supplement: Transparent reporting form [file elife-55911-transrepform.docx]

***eLife’s* transparent reporting form**

We encourage authors to provide detailed information *within their submission* to facilitate the interpretation and replication of experiments. Authors can upload supporting documentation to indicate the use of appropriate reporting guidelines for health-related research (see [EQUATOR Network](http://www.equator-network.org/%20)), life science research (see the [BioSharing Information Resource](https://biosharing.org/)), or the [ARRIVE guidelines](http://www.plosbiology.org/article/info:doi/10.1371/journal.pbio.1000412) for reporting work involving animal research. Where applicable, authors should refer to any relevant reporting standards documents in this form.

If you have any questions, please consult our Journal Policies and/or contact us: [editorial@elifesciences.org](mailto:editorial@elifesciences.org).

**Sample-size estimation**

- You should state whether an appropriate sample size was computed when the study was being designed
- You should state the statistical method of sample size computation and any required assumptions
- If no explicit power analysis was used, you should describe how you decided what sample (replicate) size (number) to use

Please outline where this information can be found within the submission (e.g., sections or figure legends), or explain why this information doesn’t apply to your submission:

We did not perform sample size calculations. The numbers of samples used (as noted in the Methods section) was based on knowledge from extensive previous genetic modifier studies in the mouse, including a conditional genetic knockout study (e.g. Kovalenko et al, PLoS One. 2012;7(9):e44273, Pinto et al. PLoS Genet. 2013 Oct;9(10):e1003930), of the number of mice needed to detect modification of the phenotypes tested. We were effectively powered, based on these studies, to detect the fairly subtle phenotype modification. In addition, with the knowledge that these phenotypes are strongly repeat length-dependent, mice in different genotype groups were controlled for inherited repeat length (Supplementary Data File 1). For the RNA-seq analyses we aimed for numbers of mice shown in a previous RNA-seq study (Langfelder et al. [Nat Neurosci. 2016 Apr; 19(4): 623–633) to exhibit extensive transcriptional dysregulation due to the HttQ111 allele.](https://www.ncbi.nlm.nih.gov/entrez/eutils/elink.fcgi?dbfrom=pubmed&retmode=ref&cmd=prlinks&id=26900923)

**Replicates**

- You should report how often each experiment was performed
- You should include a definition of biological versus technical replication
- The data obtained should be provided and sufficient information should be provided to indicate the number of independent biological and/or technical replicates
- If you encountered any outliers, you should describe how these were handled
- Criteria for exclusion/inclusion of data should be clearly stated
- High-throughput sequence data should be uploaded before submission, with a private link for reviewers provided (these are available from both GEO and ArrayExpress)

Please outline where this information can be found within the submission (e.g., sections or figure legends), or explain why this information doesn’t apply to your submission:

All replicates are biological replicates (N=individual mice as explained in the Methods, and detailed in the Figure Legends).

Mice were matched closely for inherited CAG repeat length between genotype groups in each cohort (Supplementary Data File 1) and mice with outlier CAG lengths were excluded.

A single outlier in the RNA-seq data due to technical artifact, was excluded, as described in the Methods.

RNA-seq data have been uploaded to GEO

**Statistical reporting**

- Statistical analysis methods should be described and justified
- Raw data should be presented in figures whenever informative to do so (typically when N per group is less than 10)
- For each experiment, you should identify the statistical tests used, exact values of N, definitions of center, methods of multiple test correction, and dispersion and precision measures (e.g., mean, median, SD, SEM, confidence intervals; and, for the major substantive results, a measure of effect size (e.g., Pearson's r, Cohen's d)
- Report exact p-values wherever possible alongside the summary statistics and 95% confidence intervals. These should be reported for all key questions and not only when the p-value is less than 0.05.

Please outline where this information can be found within the submission (e.g., sections or figure legends), or explain why this information doesn’t apply to your submission:

Statistical methods are described and justified in the Methods section

Where graphs are represented as mean+/-SD we are providing raw data for these figures in the Source data files.

P values with 95% CIs are reported in the text, together with the statistical test used

(For large datasets, or papers with a very large number of statistical tests, you may upload a single table file with tests, Ns, etc., with reference to sections in the manuscript.)

**Group allocation**

- Indicate how samples were allocated into experimental groups (in the case of clinical studies, please specify allocation to treatment method); if randomization was used, please also state if restricted randomization was applied
- Indicate if masking was used during group allocation, data collection and/or data analysis

Please outline where this information can be found within the submission (e.g., sections or figure legends), or explain why this information doesn’t apply to your submission:

Groups were assigned according to the genotype of the mice.

Counting of nuclear inclusions and QC steps for RNA-seq were conducted blind to genotype, as stated in the Methods.

**Additional data files (“source data”)**

- We encourage you to upload relevant additional data files, such as numerical data that are represented as a graph in a figure, or as a summary table
- Where provided, these should be in the most useful format, and they can be uploaded as “Source data” files linked to a main figure or table
- Include model definition files including the full list of parameters used
- Include code used for data analysis (e.g., R, MatLab)
- Avoid stating that data files are “available upon request”

Please indicate the figures or tables for which source data files have been provided:

Source data files are provided with the raw data for Figure1- figure supplement 1, Figure 2-figure supplement 3, Figure 6, Figure 6- figure supplement 1, Figure 6- figure supplement 2, Figure 6- figure supplement 3, Figure 8, Figure 8- figure supplement 1.

For the RNA_seq data and analyses following data and code files are included:

29_genes.txt

assessingDEGoverlapsignificance.R

cleaningP.R

compareThisStudyLangfelderStudy.R

Folder of Counts data

Folder of DEG lists

Folder of metadata files

Mus_musculus_.GRCm38.75.genes_to_remove.txt

pathwayComparison.R

Folder of Pathways data

plot_and_significance_29genes_relativeEffect.R

rescue_effect_analysis.R

runDAVID.R

runEdgeR.Langfelder.R

runEdgeR.SVA.R
